# Comparative Metagenomic and Metatranscriptomic Analyses Reveal the Response of Black Soldier Fly (*Hermetia illucens*) Larvae Intestinal Microbes and Reduction Mechanisms to High Concentrations of Tetracycline

**DOI:** 10.3390/toxics11070611

**Published:** 2023-07-13

**Authors:** Yaxin Pei, Mengxiao Sun, Jiran Zhang, Aojie Lei, Hongge Chen, Xiangtao Kang, Hongyuhang Ni, Sen Yang

**Affiliations:** 1 Key Laboratory of Agricultural Microbial Enzyme Engineering (Ministry of Agriculture), School of Life Sciences, Henan Agricultural University, Zhengzhou 450046, China; peiyx14@163.com (Y.P.); mengxiaosun@126.com (M.S.); zhangjiran@henau.edu.cn (J.Z.); aojieray@126.com (A.L.); honggeyz@henau.edu.cn (H.C.); xtkang2001@263.net (X.K.); 2 Department of Infectious Diseases and Public Health, Jockey Club College of Veterinary Medicine and Life Sciences, City University of Hong Kong, Kowloon, Hong Kong

**Keywords:** BSFL, tetracycline, intestinal microbes, functional genes, response mechanism

## Abstract

Black soldier fly (*Hermetia illucens* L.) larvae (BSFL) possess remarkable antibiotic degradation abilities due to their robust intestinal microbiota. However, the response mechanism of BSFL intestinal microbes to the high concentration of antibiotic stress remains unclear. In this study, we investigated the shift in BSFL gut microbiome and the functional genes that respond to 1250 mg/kg of tetracycline via metagenomic and metatranscriptomic analysis, respectively. The bio-physiological phenotypes showed that the survival rate of BSFL was not affected by tetracycline, while the biomass and substrate consumption of BSFL was slightly reduced. Natural BSFL achieved a 20% higher tetracycline degradation rate than the germ-free BSFL after 8 days of rearing. Metagenomic and metatranscriptomic sequencing results revealed the differences between the entire and active microbiome. Metatranscriptomic analysis indicated that *Enterococcus*, *Vagococcus*, *Providencia*, and *Paenalcaligenes* were the active genera that responded to tetracycline. Furthermore, based on the active functional genes that responded to tetracycline pressure, the response mechanisms of BSFL intestinal microbes were speculated as follows: the *Tet* family that mediates the expression of efflux pumps expel tetracycline out of the microbes, while *tetM* and *tetW* release it from the ribosome. Eventually, tetracycline was degraded by deacetylases and novel enzymes. Overall, this study provides novel insights about the active intestinal microbes and their functional genes in insects responding to the high concentration of antibiotics.

## 1. Introduction

Antibiotics are widely used in medicine, agriculture, and animal husbandry [1], with China producing more than 200,000 tons of antibiotics yearly [2]. The annual global consumption of antimicrobial drugs is also increasing and is projected to grow by 104,000 tons by 2050 [3]. However, due to the low efficiency of tetracycline biosorption by animals and humans, 50–80% of tetracycline is released into the environment, leading to the spread of multiple drug-resistance genes and significant toxicity into the ecosystem [4]. Physical and chemical techniques, such as electrochemical methods and the adsorption of porous materials, are being used to remove antibiotics in the environment [5]. However, these techniques are costly and tend to cause secondary contamination. In contrast to traditional methods, bioremediation techniques can effectively remove antibiotics [6]. Currently, many studies have been conducted to design strategies that remove antibiotics via efficient degradation of strains, including *Klebsiella* sp.TR5 [7], *Trichosporon mycotoxinivorans* XPY-10 [8], *Stenotrophomonas maltophilia* DT1 [9], *Serratia marcescens* WW1 [10], *Arthrobacter nicotianae* OTC-16 [6], and *Alcaligenes* sp. T17 [11]. However, most high-efficient antibiotic degradation microbes are pathogenic and risk spreading antibiotic resistance genes. Moreover, these strains struggle to consistently coexist with the indigenous microbial community [6,12]. Therefore, novel approaches are needed to remediate antibiotic contamination.

Recently, there has been growing interest in insects due to their potential ability to treat different types of pollutants. This ability is attributed to their wide range of feeding habits, high economic value, easy management, and low risk of antibiotic resistance gene transmission [13]. Among insects, black soldier fly larvae (BSFL), also known as *Hermetia illucens* (Diptera: Stratiomyidae), have shown high capacity to degrade most antibiotics, which is primarily credited to its powerful intestinal flora [14,15]. For instance, Yang et al. discovered that BSFL effectively degraded 84.22% of ciprofloxacin in poultry manure within 12 days [16]. Moreover, they isolated *Klebsiella pneumoniae* CIP1 and *Proteus mirabilis* CIP5, which can efficiently degrade more than 84% of 50 mg/kg ciprofloxacin in 12 days. In addition, Luo et al. achieved an 84.9% reduction in lincomycin in fermentation residue via BSFL treatment within 12 days [17]. *Acinetobacter* and *Sphingobacterium* are presumed to be the dominant groups involved in lincomycin degradation. Furthermore, Liu et al. achieved 63.3–82.7% oxytetracycline degradation at an initial concentration of 100–3042.3 mg/kg in 7–8 days after BSFL treatment [5,18]. Moreover, they identified the remarkable alteration of the relative abundances of *lgnatzschineria*, *Enterococcus*, and *Providencia*.

To discover the main functional genes, molecular methods were used to detect the abundance/expression level of related functional genes in previous works. For instance, Luo et al. explored the expression levels of six lincomycin resistance genes (*lnuA*, *lnuB*, *lnuC*, *lnuD*, *lnuE*, and *lnuF*) via qPCR [17]. Similarly, Liu et.al selectively identified 180 or 285 ARGs in the gut microbial of BSFL under the pressure of oxytetracycline using high-throughput qPCR (HT-qPCR) [5,18]. The results clearly showed that the abundances of *tetA*, *tetB*, and *tetC* increased. However, almost all existing studies on the BSFL gut flora and functional genes that respond to tetracycline were based on a combination of MiSeq and qRT-PCR analyses, which are limited in providing a comprehensive understanding of the overall environment in terms of the microbial community and all of its functional genes changes.

Metagenomic analysis is a field of study that examines the genome DNA (gDNA) of environmental microbial communities, revealing the functional genes that are present in these communities [19]. In contrast to metagenomics, metatranscriptomics analyzes the mRNA of microbial communities in the environment to reveal the active micro-organisms and their functional gene resources that respond to changes in environmental factors [20]. However, there is still a lack of studies related to the changes in BSFL gut flora composition and functional genes recorded via metagenomic and metatranscriptomic analysis techniques. Furthermore, previous work mainly focused on the degradation of BSFL to low concentrations of antibiotics. The effects of high concentrations of antibiotics on gut microbes and functional genes of BSFL are unclear.

In this study, we aimed to explore the changes in gut microbial community structure and functional genes at the genomic and transcriptomic levels in response to high tetracycline stress in BSFL via combined comparative metagenomic and metatranscriptomic analyses. The objectives of the study included determining the changes in the physiological characteristics of BSFL and the gut microbial community in response to high tetracycline presence, as well as the changes in the abundances of associated antibiotic resistance genes and degradation genes, thus inferring potential mechanisms of antibiotic degradation used by BSFL. The findings of this study provide a theoretical basis for the application of BSFL to dispose of tetracycline-contained contaminants.

## 2. Results

### 2.1. Effect of Tetracycline on the BSFL Growth

**Figure 1:**
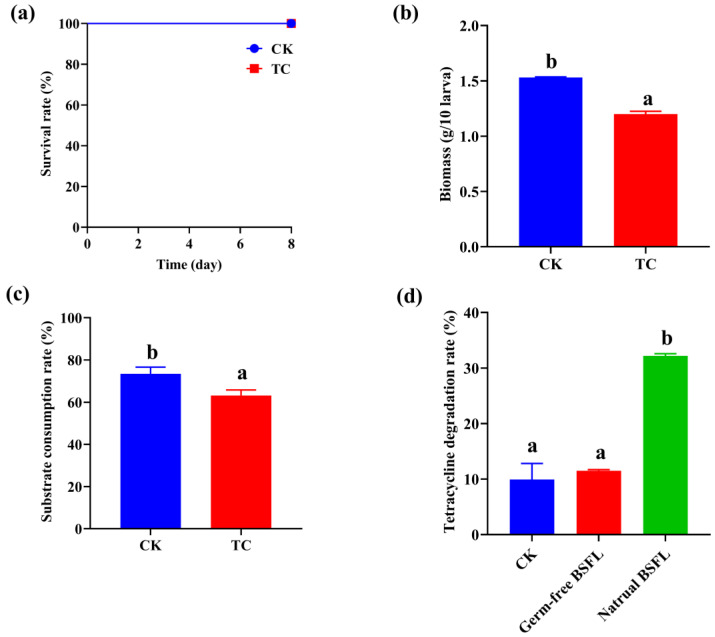
The bio-physiological parameters’ (**a**) survival rate, (**b**) Biomass (g/10 larva), (**c**) substate consumption rate of BSFL in the CK and TC groups, and (**d**) tetracycline degradation efficiency in the CK, germ-free, and natural BSFL groups. Data are presented as mean ± standard deviation. Values with different letters mean significant differences at *p* < 0.05, as determined via Tukey’s test.

To assess the effect of tetracycline on the growth and substrate utilization of BSFL, a high concentration of tetracycline (1250 mg/kg) was added to the larvae’s diet. The results showed that tetracycline did not affect the survival of BSFL during the 8-day feeding time (Figure 1a). In addition, after 8 days of rearing, the BSFL experienced a modest decrease in biomass and substrate consumption rate of 1.20 g/10 larva and 63.2%, respectively, compared to the control group without antibiotic supplementation (1.53 g/10 larva and 73.5%) (Figure 1b,c). Meanwhile, the natural BSFL group achieved a tetracycline degradation rate of 32.21%, which was far higher than those of CK (9.92%) and the germ-free model groups (11.51%) (Figure 1d). These results indicated that BSFL has excellent tolerance of the high concentration of tetracycline, and its intestinal flora plays an essential role in the tetracycline degradation process.

### 2.2. Overview of Metagenomic and Metatranscriptomic Raw Data

Metagenomic and metatranscriptomic sequencing methods were used to analyze the microbial community and functional genes involved in the process of tetracycline degradation. Raw reads of 113,499,814, 121,370,872, 115,167,960, and 117,241,914 were obtained for the CK-MG, TC-MG, CK-MT, and TC-MT groups, respectively. The sequencing data generated 41,586, 43,057, 9526, and 2500 contigs from 78.55%, 85.77%, 92.77%, and 80.61% clean reads, respectively. Additionally, the ratio of clean bases to raw bases for each group was 77.91%, 85.09%, 92.20%, and 80.20%, respectively. Clean reads Q20 and Q30 represent the percentage of bases with a Phred value of >20 or 30. The Q20 and Q30 statistics in all samples exceeded 97.4% and 94.0%, respectively (Table 1). The N50 and N90 for MG data were ≥4314 bp and 650 bp, respectively, while for MT data, they were ≥1078 and 565 bp, respectively. All of the above results implied that the MG and MT data were of the high sequencing quality required for further analysis.

### 2.3. Composition and Diversity of Microbial Communities in BSFL Gut

**Figure 2:**
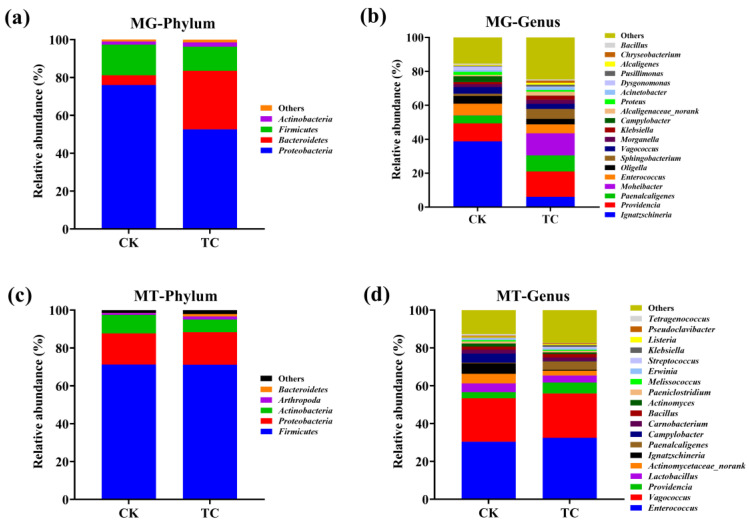
BSFL intestinal microbial composition at (**a**) phylum and (**b**) genus levels based on metagenomic sequencing data; (**c**) phylum and (**d**) genus levels based on metatranscriptomic sequencing data in CK and TC groups.

Metagenomic sequencing data revealed that the microbial community structure was largely changed at the phylum level in TC group compared to CK group. *Proteobacteria* was the most dominant phylum, having a relative abundance of 75.98% and 52.65% in the CK and TC groups, respectively, followed by *Bacteroidetes* and *Firmicutes* (Figure 2a). Notably, the relative abundance of *Bacteroidetes* increased from 5.16 to 30.84% under the stress of tetracycline. At the genus level, the most abundant microbes remained the same, but their abundances showed considerable distinctions between the TC and CK groups. *Ignatzschineria* and *Providencia* were the most abundant genera in the CK and TC groups, accounting for 38.78% and 14.98%, respectively (Figure 2b). Meanwhile, the addition of tetracycline increased the percentage of *Moheibacter* and *Sphingobacterium* from 0.10 to 13.07% and from 1.35 to 5.89%, respectively. These results indicated that the indigenous intestinal microbiome of BSFL was influenced by tetracycline.

The results of metatranscriptomic analysis were markedly different from the metagenomic results. *Firmicutes* was the most dominant phylum, having a richness of 71.20% and 71.10% in CK and TC groups, respectively (Figure 2c), followed by *Proteobacteria* and *Actinobacteria*. The relative abundance of *Actinobacteria* was decreased from 9.76% in the CK group to 6.70% in the TC group. At the genus level, the relative abundances of the dominant microbes, including *Enterococcus*, *Vagococcus*, *Providencia*, and *Paenalcaligenes*, were increased with TC treatment (Figure 2d). The above results demonstrate the differences between the total and active microbes in BSFL intestine.

### 2.4. Antimicrobial Resistance (AMR) Gene Analysis

**Figure 3:**
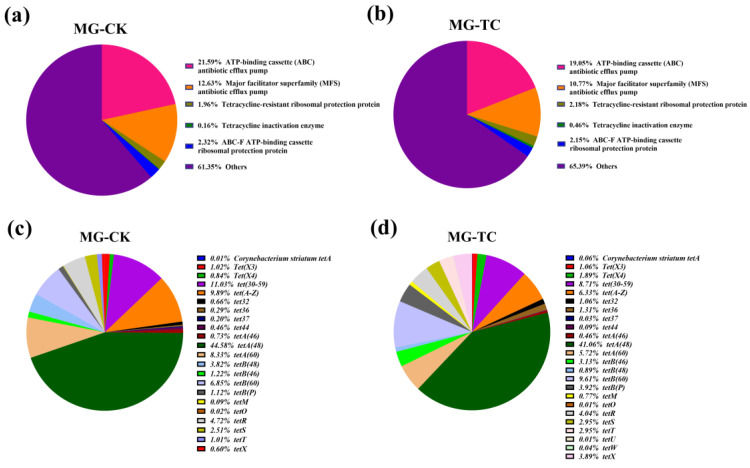
Abundances of antibiotic resistance gene (AMR) types in the (**a**) CK and (**b**) TC groups. Abundances of *Tet* family specific genes in the (**c**) CK and (**d**) TC groups based on metagenomic sequencing data.

Functional genes of BSFL intestinal microbes at genetic and expressed levels were revealed via annotation of resistance genes in the CARD database. At the DNA level, CK and TC groups contained 707 and 711 AMR genes, respectively. The overall effect of tetracycline on the types and abundances of antibiotic resistance genes in BSFL gut microbes was small (Figure 3a–d). Specifically, the abundances of genes associated with ATP-binding cassette (ABC) antibiotic efflux pump and major facilitator superfamily (MFS) antibiotic efflux pump were slightly decreased, whereas the abundances of genes related to tetracycline-resistant ribosomal protection protein and tetracycline inactivation enzyme were slightly elevated by 0.22% and 0.30%, respectively (Figure 3a,b). The abundance of the *tetA(48),* which was the most abundant tetracycline resistance gene in both groups, decreased from 44.58% in the CK group to 41.06% in the TC group (Figure 3c,d). Moreover, the abundance of *tetB(48)* was decreased from 3.82 to 0.89%, whereas the abundances of *tetB(60)* and *tet(X)* were elevated from 6.85% and 0.60% to 9.61% and 3.89%, respectively, after tetracycline treatment. These findings suggest that tetracycline has a minor influence on the abundance of tetracycline resistance genes in BSFL gut microbes.

**Figure 4:**
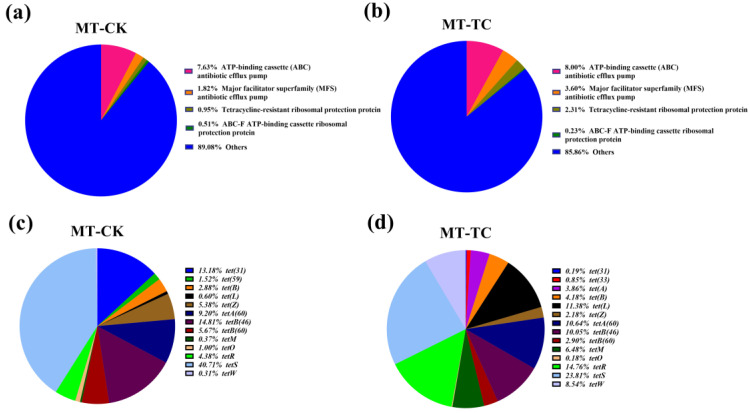
Abundances of antibiotic resistance gene (AMR) types in the (**a**) CK and (**b**) TC groups. Abundances of *Tet* family specific genes in the (**c**) CK and (**d**) TC groups based on metatranscriptomic sequencing data.

In addition, changes in antibiotic resistance genes at the transcriptional level were analyzed. The CARD database showed that 153 and 118 AMR genes were detected at the RNA level in the CK and TC groups, respectively. Moreover, the genes’ expression of ABC antibiotic efflux pump, MFS antibiotic efflux pump, and tetracycline-resistant ribosomal protection protein were upregulated from 7.63%, 1.82%, and 0.95% to 8.00%, 3.60%, and 2.31%, respectively (Figure 4a,b). However, for tetracycline inactivation enzymes, the *tet(X)* family was not annotated. These results indicated that there must be other enzymes in the BSFL gut that presented as tetracycline-degrading enzymes. Furthermore, the analyses of the tetracycline resistance genes revealed that *tet(31)*, *tetA(60)*, *tetS*, and *tetB(46)* were the most dominant genes in CK group, with expression levels decreasing to 0.19%, 10.64%, 23.81%, and 10.05% in TC group. Further, the abundances of *tet(33)*, *tet(A)*, *tet (B)*, *tet(L)*, *tetA(60)*, *tetR*, *tetM*, *tetW,* and *tet(L)* were increased in the TC group compared to the CK group (Figure 4c,d). The above results suggested that tetracycline has effects on the expression of the genes of the overall BSFL gut microbes. Further, these results also indicate that genes that respond to antibiotic stress in the BSFL gut flora vary greatly in terms of the copy numbers and expression levels.

### 2.5. Differentially Expressed Genes in Metatranscriptomic Library

**Figure 5:**
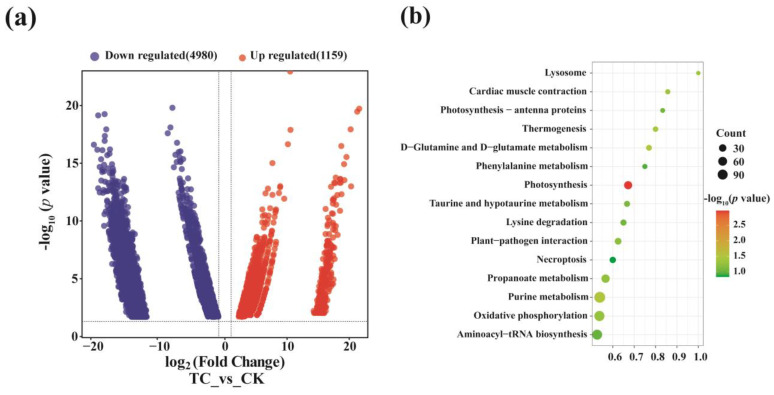
Differential gene expression analysis between the TC and CK groups. (**a**) Differences in gene expression profiles; (**b**) the top 15 KEGG enrichment terms of differentially expressed genes.

To further investigate the influence of tetracycline on the expression of all genes in the BSFL intestinal microbe, the differentially expressed genes between two groups were analyzed. It showed that 1159 genes were upregulated, whereas 4980 genes were downregulated, under tetracycline stress (Figure 5a). KEGG enrichment analysis showed that among the top 15 functional gene enriched pathways, D-Glutamine and D-glutamate metabolism underwent significant upregulation (*p* = 0.03), with the gene ratio reaching 0.77 (Figure 5b), which is the only ratio reported that is related to tetracycline resistance [21].

### 2.6. Potential Tetracycline Degradation Genes

**Figure 6:**
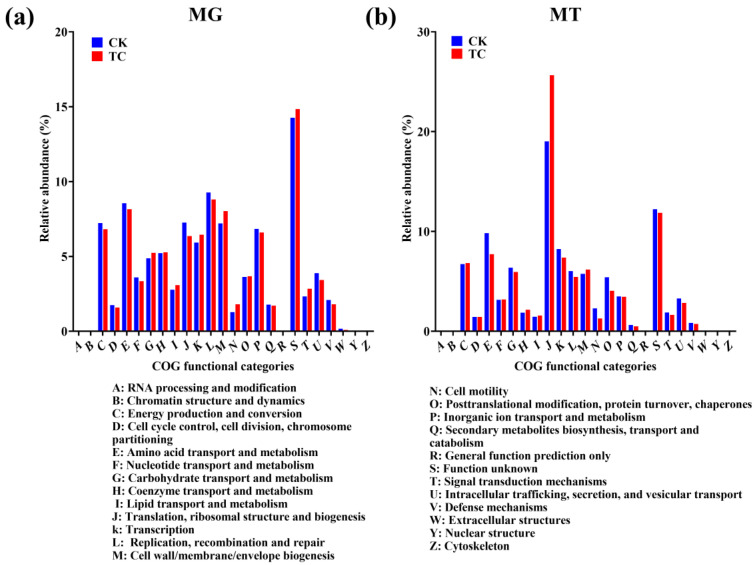
Distribution of COG functional categories in the CK and TC groups based on (**a**) metagenomic and (**b**) metatranscriptomic sequencing data.

The COG database was utilized to annotate the functional genes to analyze potential tetracycline degradation-related genes. The metagenomic data revealed that the diversity and abundances of functional genes in the overall BSFL intestinal microbiota remained unchanged under tetracycline pressure (Figure 6a). In contrast, metatranscriptomic data exhibited that genes belonging to the functional category of “translation, ribosomal structure and biogenesis” increased from 19.02% (CK) to 25.66% (TC) (Figure 6b). This result indicated that tetracycline has an obvious effect on the translation and expression of genes, and the upregulated genes are likely to be related to the degradation of tetracycline. Further, the above results implied that the functional genes of BSFL gut exhibited a positive transcriptional response to tetracycline, but did not cause any changes in their gene copy numbers.

**Figure 7:**
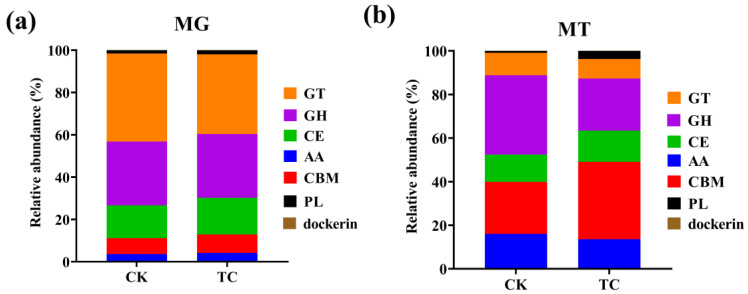
The relative abundances of genes that encode different CAZy classes (GTs: Glycosyl Transferases; GHs: Glycoside Hydrolases; CEs: Carbohydrate Esterases; AAs: Auxiliary Activities; CBMs: Carbohydrate-Binding Modules; PLs: Polysaccharide) in the CAZy database at level 1 in the CK and TC groups based on (**a**) metagenomic and (**b**) metatranscriptomic sequencing data.

The distribution of functional genes based on the CAZy database was similar to that of the COG analysis. There was a remarkable difference in the abundances of annotated carbohydrates between the genomic and transcriptomic levels. Further, while functional gene distribution was not distinctly different between the two groups at the genomic level, it showed quite differences at the transcriptomic level (Figure 7a,b). At the DNA level, glycosyl transferases (GTs, 37.68–41.64%) and glycoside hydrolases (GHs, 30.08–31.14%) were the most two dominant groups, followed by carbohydrate esterases (CEs, 15.57–17.31%) and carbohydrate-binding modules (CBMs, 7.54–8.71%) (Figure 7a). In contrast, the gene abundances of both CEs and CBMs were increased at the transcriptional level, from 12.51 to 14.26% and from 23.91 to 35.55%, respectively, while GTs, GHs, and AAs decreased in the TC group in comparison to the CK group (Figure 7b). These results illustrate that tetracycline affects not only the abundances of these genes, but also their expression level.

Tet (X), horseradish peroxidase, manganese peroxidase, glutathion S-transferase, lignin peroxidase, and laccase are known as the tetracycline degradation enzymes, and their corresponding genes were identified via metagenomic and metatranscriptomic libraries, respectively. Genes involved in antibiotic degradation, specifically AA&CE, were annotated and are presented in Table 2. Notably, AA1 (laccase), AA2 (manganese peroxidase), AA7 (esterase), and AA10 (monooxygenases), which have been reported to be involved in tetracycline degradation, were identified in the metagenomic database, but were not identified in the metatranscriptomic database. This result suggested that the efficient tetracycline degradation ability of the BSFL gut microflora was not caused by known tetracycline-degrading enzymes. Therefore, we speculated that novel and efficient tetracycline-degrading enzymes may exist in the BSFL gut microflora. Moreover, CEs that were in charge of degrading other antibiotics presented distinctive differences between metagenomic and metatranscriptomic levels. The expression of genes corresponding to CE1, CE3, CE4, CE6, CE7, CE8, and CE11 was upregulated after tetracycline treatment (Table 2), which indicated that they may be involved in tetracycline degradation.

### 2.7. Possible Mechanisms of BSFL Intestinal Microbiome Response to High Tetracycline Concentration Stress

**Figure 8:**
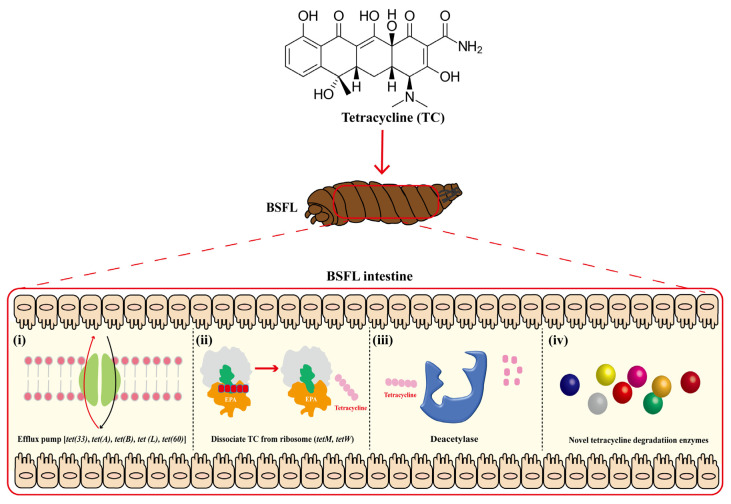
Speculated tetracycline response mechanisms of BSFL’s intestinal microbiota. (**i**) Efflux pump, (**ⅱ**) Dissociate TC from ribosome, (**ⅲ**) Deacetylase, and (**ⅳ**) Novel tetracycline degradation enzymes.

Under high levels of TC stress, the BSFL’s active gut microbial community structure and expression of related functional genes were changed. Based on the results presented above, mechanisms of the BSFL intestinal microbiota response to the high tetracycline concentrations were supposed, as shown in Figure 8. Firstly, *Tet* family genes, including *tet(33), tet(A), tet(B)*, *tet(L)*, and *tetA(60),* act as efflux pumps to remove tetracycline from the cell. In addition, *tetM* and *tetW* may dissociate tetracycline that has already bound to the ribosome, thus promoting the diffusion of tetracycline in microbes, as well as accelerating subsequent degradation. Notably, other known tetracycline-degrading enzyme genes, including Tet(X), horseradish peroxidase, manganese peroxidase, lignin peroxidase, glutathione-S transferase, and laccase, were not detected at the transcriptomic level. Deacetylase (CE3, CE4, CE7, CE8, and CE11) was identified as the only type of tetracycline-degrading enzyme used in this study, which assisted in the hydrolysis of tetracycline and, ultimately, its removal. Therefore, it is concluded that there may be novel and highly efficient tetracycline-degrading enzymes present in the BSFL’s intestinal microbes.

## 3. Discussion

BSFL has been utilized for the degradation of various antibiotics [17,22,23]. In this study, we investigated the ability of BSFL to withstand and degrade a high concentration of exogenous tetracycline (1250 mg/kg). The results showed that BSFL maintained a 100% survival rate for 8 days after exposure to the high concentration of tetracycline (Figure 1a), indicating their strong ability to resist this antibiotic. Although the biomass and substrate consumption rate decreased by only 0.33 g/10 larva and 10%, respectively (Figure 1b,c), the BSFL intestinal microbiome still demonstrated a significant ability to degrade tetracycline. Intestinal flora assisted BSFL in achieving a tetracycline degradation rate of 32.21%, which was higher than that of the germ-free group (11.51%) (Figure 1d). This result contrasts with those of a previous study by Cai et al., which showed that normal BSFL achieved a 95.5–96.9% degradation rate at low initial concentrations of 20–80 mg/kg tetracycline, while sterile BSFL reduced tetracycline content by ~60% [22]. The difference between each study’s results could be due to the fact that the high tetracycline concentration inhibits the activity of some micro-organisms, thus increasing the time required to achieve a higher degradation rate. Moreover, Liu et al. found that BSFL could degrade substrates containing oxytetracycline (initial concentration of 434.4 mg/kg) by 65.9% within 8 days [5]. These results suggest that BSFL can effectively degrade high concentrations of tetracycline, with the assistance of BSFL intestinal flora, without affecting their own growth and survival.

The composition of the gut microbiota at the phylum level revealed that *Proteobacteria*, *Bacteroidetes*, and *Firmicutes* were the dominant microbes at the genetic level (Figure 2a), which is consistent with a previous study that found that these three phyla account for more than 99% of the BSFL’s gut microbial community [22]. Moreover, Yun discovered that *Proteobacteria* and *Firmicutes* constitute a major part of the gut microbiota of 218 different insect species [24]. After feeding the tetracycline-containing substrate for 8 days, the relative abundances of *Proteobacteria* and *Firmicutes* in the BSFL gut flora decreased by 23.33% and 3.39%, respectively, while that of *Bacteroidetes* increased by 25.68%. The results of this study were in accordance with a previous study’s gDNA-based Miseq sequencing results [22]. This consistency indicates that gDNA-based analysis tools differ only slightly in terms of the relative abundances of different phyla, but not in terms of the composition and abundance changes in the microbial community.

Interestingly, at the transcriptional level, the microbial structure presented differently to that at the genomic level, suggesting differences in the composition of the active and entire microbiomes. Furthermore, the microbial community did not show huge changes between two groups. In both the CK and TC groups, *Firmicutes* was the dominant phylum, followed by *Proteobacteria*, *Actinobacteria*, *Arthropoda*, and *Bacteroides* (Figure 2c). Previous studies indicated that *Firmicutes* and *Proteobacteria* carry a variety of tetracycline resistance genes [25]. The relative abundance of *Actinobacteria* was decreased, while that of *Bacteroides* was increased. This result is consistent with a study that shows a strong correlation between *Bacteroides* and the degradation of tetracycline in disposed pig manure by housefly larvae [26]. Ji et al. also discovered that the relative abundance of *Bacteroides* in the human intestine rose from 1.68% to 5.70% under tetracycline exposure [27].

At the genus level, the dominant microbes in BSFL gut also presented differently based on gDNA and mRNA analysis (Figure 2b,d). The relative abundances of active genera, including *Enterococcus*, *Providencia*, *Paenalcaligenes*, and *Carnobacterium*, was increased via tetracycline treatment (Figure 2d), which indicated that they may correlate with the tetracycline-degrading ability of BSFL. *Enterococcus* is frequently isolated from tetracycline-contaminated environments and considered to be a reservoir of tetracycline and macrolide–lincosamide–streptogramin (MLS) resistance genes [28]. *Providencia is* one of the core bacteria in the BSFL gut and believed to be responsible for the tetracycline-modifying gene *tetX* [29]. *Paenalcaligenes* were also reported to strongly respond to antibiotic exposure [30]. Interestingly, *Carnobacterium* was not previously known to possess resistance to tetracycline [31]. This shift may be due to gene horizontal transfer, which allowed it to acquire the tetracycline resistance genes.

Similar to the results of structural analysis of the BSFL gut microbiota, their functional genes differed distinctly between the transcriptomic and genomic levels (Figure 3 and Figure 4). Metatranscriptomic analysis provided a comprehensive understanding of the whole gene expression and regulation of BSFL gut microbiome in response to tetracycline. Firstly, the KEGG annotation results showed a significant upregulation of genes associated with D-Glutamine and D-glutamate metabolism under tetracycline stress (Figure 5b). Zhao et al. reported that glutamine modulates the permeability of bacterial cell membranes, which, in turn, affects the amount of antibiotics that enter the bacteria [21]. Furthermore, the CARD database comparison results illustrated the expression differences of antimicrobial resistance genes before and after tetracycline treatment. Genes that express ABC-binding antibiotic efflux pump, major facilitator superfamily antibiotic efflux pump, and tetracycline-resistant ribosomal protein families were also upregulated (Figure 4a,b). The efflux pump is one of the main means through which bacteria develop drug resistance [32]. Notably, *Tet* family genes, including *tet(33)*, *tet(A)*, *tet(B)*, *tet(L)*, *tetA(60)*, *tetR*, *tetM*, and *tetW*, showed distinct differences at expressed levels (Figure 4c,d). Among these genes, *tet(33), tet(A), tet(B)*, *tet(L)*, and *tetA(60)* are mainly involved in the expression of the efflux pump [33,34]. *TetR* is a repressor of the tetracycline resistance element, the N-terminal region of which forms a helix-turn-helix structure that binds DNA. Binding of tetracycline to *tetR* reduces the repressor’s affinity for the *tetA* promoter–operator sites [35]. *tetM* was reported to induce the translocation of ribosome-bound tetracycline to the free state and shorten the half-life of the drug in most gram-positive bacteria [36]. *TetW* was found to be widespread in the BSFL gut community in the previous study, which blocked the action of tetracycline through ribosome non-covalent modifications [22,37]. Meanwhile, we were surprised to find that *tet(X)*, which has tetracycline degradation ability, was not expressed in either the CK or TC group, which is in contrast to the metagenomic results. Therefore, we speculated that the BSFL’s intestinal microbiota selectively express certain genes to maintain a balance among them in order to resist the external high concentration of tetracycline stimulation.

Drug resistance genes are not the only factors that aid the host in resisting tetracycline stress, as some proteins with hydrolytic and redox functions may also be involved in tetracycline degradation. The differentially expressed genes annotated in the COG database that illustrated active functional genes under the “Translation, ribosomal structure and biogenesis” category were elevated with tetracycline treatment (Figure 6b). This observation is in line with a previous study by Liu et. al., which used a knockout approach to find that translation elongation factor 4 (LepA) affects the ribosome biogenesis of cells under the stress of tetracycline [38]. Results of the CAZy database annotation showed that genes that enable tetracycline-degrading enzymes that were present under the AA module were detected at the metagenomic level (Figure 7). Specifically, AA1 gene family that annotated for laccase, and AA2 gene family that annotated for manganese peroxidase and lignin peroxidase, were identified. All three types of protein were previously reported to possess the ability to degrade tetracycline [12,39,40,41,42]. However, no regulatory changes in the genes under the AA module occurred at the RNA-seq level. This result suggests that there may be other metabolic pathways involved in tetracycline degradation.

In this study, seven annotated gene families (CE1, 3–4, 6–8, 11) were found to be upregulated after tetracycline treatment (Table 2). CEs were annotated as esterase, which were reported to degrade β-lactam and macrolide antibiotics by hydrolyzing ester bonds [43]. This finding suggests that a correlation exists between different antibiotic resistance levels. Meanwhile, deacetylases classified as CE3, CE4, CE7, CE8, and CE11 deserve our attention due to their roles in the hydrolysis of acetamides [44], which may be involved in tetracycline degradation via deamination. Furthermore, 171 unknown function genes were upregulated in our study and may be involved in tetracycline degradation. Therefore, the subsequent study will be focused on exploring the unknown efficient tetracycline-degrading enzymes.

## 4. Conclusions

This study employed a combined comparative metagenomic and metatranscriptomic approach to investigate alterations in the BSFL intestinal microbiome under a high concentration of tetracycline pressure. BSFL achieved an impressive 32.21% tetracycline reduction in 8 days under the 1250 mg/kg tetracycline concentration. Through microbiome sequencing, we discovered that tetracycline does indeed affect the overall ecological community’s structure and abundances in the gut, though the composition of active genera at the transcriptional level remained largely unchanged. Through the annotation of the functional genes in several databases, such as eggNOG, CAZy, and CARD, the abundance changes in potential active tetracycline-degrading genes in the BSFL gut microbiome were examined. Further, the superior tetracycline resistance mechanisms of BSFL gut microbes have been confirmed. The findings of this study provide a valuable reference for future investigations into insects’ in situ bioremediations of antibiotic contamination. Our future research will be focused on exploring the novel and highly efficient tetracycline degradation enzymes.

## 5. Materials and Methods

### 5.1. BSFL Husbandry and Substrate

The BSFL utilized in this study were acquired from the culturing center of Henan Agriculture University. The 3rd instar larvae were reared at a moisture content of 70% and a temperature of 30 °C based on the protocol established in our previous study [45]. Next, they were divided into two groups, including the CK (fed soybean meal only) and TC (fed soybean meal containing a final concentration of 1250 mg tetracycline per kg dry weight) groups. Deionized water was added to the substrates daily to maintain a moisture content close to 70%. The tetracycline used in this study was purchased from MCE (10 g, CAS No.: 60-54-8; MedChemExpress, Monmouth Junction, USA).

The survival rate, biomass, and substrate consumption rate were determined as follows:

BSFL’s survival rate (%) = (N_2_/N_1_) × 100%, where N_1_ and N_2_ are the initial and final number of BSFL in each group, respectively. Ten larvae were randomly selected and weighed on the 8th day of rearing to determine their biomass.
BSFL’s substrate consumption rate (%) = [(W_1_ − W_2_)/W_1_] × 100%, where W_1_ and W_2_ denote the initial and final dry weights of the wheat bran substrate during 8 days of rearing, respectively.

### 5.2. Germ-Free and Sterile BSFL Model Construction

The effect of tetracycline on the growth of larvae and the ability of non-sterile BSFL to degrade tetracycline was investigated as follows: A germ-free BSFL model was constructed according to our previous research [45]. Fresh BSFL eggs weighing approximately 0.4 g were washed with 1 mL of 2.7% NaClO and 1 mL of Sporgon solution (Beijing Mingyangkehua Bio-Technology Co. Ltd., Beijing, China) and rinsed with sterile water to obtain germ-free BSFL. The sterilized eggs were inoculated with brain heart infusion (BHI) medium and further incubated at 37 °C for 24 h. The cultures were subsequently spread on BHI agar plates to verify the sterilization effect.

Autoclaved wheat bran was moistened with 100 mL of distilled H_2_O containing tetracycline to achieve a final concentration of up to 1250 mg/kg of dry weight. Approximately 50 germ-free or sterile BSFL were introduced into each flask via the following treatments: (i) tetracycline-spiked substrate and non-sterile larvae, (ii) tetracycline-spiked substrate and sterile larvae, and (iii) tetracycline-spiked substrate and no larvae. The experiments were carried out over 8 days, during which a 1-gram sample of the substrate was collected daily and stored at −20 °C to measure the concentration of tetracycline.

### 5.3. Tetracycline Determination

Tetracycline concentration was determined using the HPLC method. Samples underwent freeze-drying, crushing using a ceramic mortar, and screening through a 100-mesh sieve. Subsequently, 1 g of sample from each group was mixed with 100 μL of the internal standard solution (200 mg/L tetracycline) in a centrifuge tube. Next, 13 mL of phosphate buffer (pH = 3) and 2 mL of methanol solution were added into the tubes. After 1 min of vertexing, the tubes were sonicated for 20 min and centrifuged at 7500 rpm for 1 min. These steps were repeated 3 times to collect the supernatant, which was subsequently filtered via a 0.22-micrometer filter [22]. A high-performance liquid chromatograph (Agilent Technologies) equipped with an octadecyl X-Terra^®^ C18 hybrid silica column (250 mm × 4.6 mm, 5 μm) was used to analyze the concentration of tetracycline. A series of tetracycline concentrations (0, 0.1, 0.5, 1, 2.5, 5 mg/L) were diluted from the standard solution (500 mg/L tetracycline) and used to draw a standard curve. The aqueous phase was composed of sodium EDTA (0.025 mol/L), calcium chloride (0.035 mol/L), and sodium acetate (0.075 mol/L) with a 7.0 pH value; methanol:acetonitrile (75:25, *v*/*v*) was used as the organic phase. The UV detection wavelength and flow rate were 355 nm and 0.8 mL/min, respectively, with the HPLC column temperature set at 27℃. The injection volume was 50 μL [6].

### 5.4. DNA and RNA Extraction, Library Construction, Sequencing

E.Z.N.A Soil Kit (Omega Bio-Tek, Norcross, GA) was used to extract gDNA from BSFL intestinal microbiota following the manufacturer’s instructions. The RNA of BSFL intestinal microbiota were extracted using our previously developed thiocyanate-high EDTA method [46]. A NanoDrop ND-2000 spectrophotometer (Thermo Fisher, MA, USA) and agarose gel electrophoresis were first used to determine the quantity and quality of gDNA/RNA. Six replicates of the CK and CR groups were mixed as one sample and then sent to Biozeron Ltd. (Biozeron, Shanghai, China) for the subsequent library construction and meta-sequencing via the Illumina Novaseq 6000 sequencing platform (Illumina, San Diego, CA, USA).

### 5.5. Data Sequencing Analysis

Raw data were extracted via the following steps to obtain a clean read dataset: (i) Trimmomatic (version0.35, available at https://github.com/usadellab/Trimmomatic) (accessed on 20 January 2022) was used to trim raw reads; (ii) reads with low-quality scores (<20) and short lengths (≤75 bp) were trimmed; (iii) Kraken 2 software annotated the species’ taxonomic data from clean reads; (iv) For RNA samples, the removal of rRNA contamination was achieved by comparing the data to the SILVA LSU (23 S/28 S) and SILVA SSU (16 S/18 S) databases via SortMeRNA software (version 2.1) (http://bioinfo.lifl.fr/RNA/sortmerna/, accessed on 20 January 2022).

Obtained clean reads were then assembled de novo using Megahit (http://github.com/voutcn/megahit, accessed on 22 January 2022). The scaffolds were then broken into contigs without gaps. Contigs of >500 bp in length were used for gene prediction. METAProdigal software (http://prodigal.ornl.gov/, accessed on 23 January 2022) was used to annotate the open reading frames (ORFs) of each sample, and CD-HIT software (http://www.bioinformatics.org/cd-hit/, accessed on 23 January 2022) was used to construct a non-redundant gene catalog with parameters set at 90% coverage and 95% consistency [47]. Next, all clean reads were further analyzed for taxonomic and functional annotations using various databases. The BLAST (BLAST Version 2.9.0+, http://blast.ncbi.nlm.nih.gov/Blast.cgi, accessed on 26 January 2022) search of each unigene was conducted against the NCBI-NR, Kyoto Encyclopedia of Genes and Genomes (KEGG, http://www.genome.jp/kegg/, accessed on 26 January 2022), Non-supervised Orthologous Groups (eggNOG, http://www.ncbi.nlm.nih.gov/COG/, accessed on 26 January 2022), Comprehensive Antibiotic Resistance Database (CARD, https://card.mcmaster.ca/, accessed on 26 January 2022), Carbohydrate-Active EnZymes (CAZy, http://www.cazy.org/, accessed on 26 January 2022) and other databases to predict gene functions and metabolic pathways.

### 5.6. Data Availability

The datasets containing metagenomic and metatranscriptomic data were deposited into the NCBI Sequence Read Archive (SRA) using accession numbers PRJNA908247 and PRJNA908248, respectively.

### 5.7. Statistical Analysis

Statistical data were analyzed via GraphPad Prism software (8.3.0). One-way ANOVA analysis of variance was used to calculate the characterization differences in all groups. *P* < 0.05 was considered statistically significant.

## 6. Patents

Two patents were generated during the process of carrying out this work. The patent numbers are as follows: CN 02210699597.8 and CN202210699598.2.

## Figures and Tables

**Table 1:** Metagenomics and metatranscriptomics sequencing data.

Samples	CK-MG	TC-MG	CK-MT	TC-MT
Raw Reads	113499814	121370872	115167960	117241914
Raw Bases	17024972100	18205630800	17275194000	17586287100
Clean Reads	89154368	104098312	106840552	94504924
Clean Bases	13264807930	15490976419	15927374626	14103693095
Contigs	41,586	43,057	9526	2500
Clean Reads in Raw Reads (%)	78.55%	85.77%	92.77%	80.61%
Clean Bases in Raw Bases (%)	77.91%	85.09%	92.20%	80.20%
Q20	97.63%	97.48%	98.25%	98.73%
Q30	95.23%	94.89%	94.06%	95.60%

**Table 2:** Abundance values of partial carbohydrate-active enzymes (AA&CE) in the CK and TC groups.

Family	CK-MG	TC-MG	CK-MT	TC-MT	Description
AA1	0.50222	1.76443	-	-	Laccase/p-diphenol:oxygen oxidoreductase/ferroxidase (EC 1.10.3.2); ferroxidase (EC 1.10.3.-); Laccase-like multicopper oxidase (EC 1.10.3.-)
AA2	2.22735	9.84698	-	-	Manganese peroxidase (EC 1.11.1.13); versatile peroxidase (EC 1.11.1.16); lignin peroxidase (EC 1.11.1.14); peroxidase (EC 1.11.1.-)
AA3	334.669	310.651	177.314	153.316	Cellobiose dehydrogenase (EC 1.1.99.18); glucose 1-oxidase (EC 1.1.3.4); aryl alcohol oxidase (EC 1.1.3.7); alcohol oxidase (EC 1.1.3.13); pyranose oxidase (EC 1.1.3.10)
AA4	189.943	294.33	-	-	Vanillyl-alcohol oxidase (EC 1.1.3.38)
AA6	199.316	380.512	4541.03	4518.02	1,4-benzoquinone reductase (EC. 1.6.5.6)
AA7	65.8805	125.302	-	-	Glucooligosaccharide oxidase (EC 1.1.3.-); chitooligosaccharide oxidase (EC 1.1.3.-)
AA10	75.5844	146.551	-	-	AA10 (formerly CBM33) proteins are copper-dependent lytic polysaccharide mono-oxygenases (LPMOs); some proteins have been shown to act on chitin, while others act on cellulose.
AA12	2.68267	5.62463	-	-	The pyrroloquinoline quinone-dependent oxidoreductase activity was demonstrated as present for the CC1G_09525 protein of *Coprinopsis cinerea*.
CE1	1464.2	1816.33	864.206	910.166	Acetyl xylan esterase (EC 3.1.1.72); cinnamoyl esterase (EC 3.1.1.-); feruloyl esterase (EC 3.1.1.73); carboxylesterase (EC 3.1.1.1); S-formylglutathione hydrolase (EC 3.1.2.12)
CE2	37.8945	30.416	-	-	Acetyl xylan esterase (EC 3.1.1.72).
CE3	180.225	156.923	25.0276	29.0947	Acetyl xylan esterase (EC 3.1.1.72).
CE4	501.018	768.334	1443.96	1964.17	Acetyl xylan esterase (EC 3.1.1.72); chitin deacetylase (EC 3.5.1.41); chito-oligosaccharide deacetylase (EC 3.5.1.-); peptidoglycan GlcNAc deacetylase (EC 3.5.1.-); peptidoglycan N-acetylmuramic acid deacetylase (EC 3.5.1.-).
CE5	0.96599	3.93697	-	-	Acetyl xylan esterase (EC 3.1.1.72); cutinase (EC 3.1.1.74)
CE6	39.7137	77.6012	133.101	948.039	Acetyl xylan esterase (EC 3.1.1.72).
CE7	61.6056	177.511	69.4047	81.6384	Acetyl xylan esterase (EC 3.1.1.72); cephalosporin-C deacetylase (EC 3.1.1.41).
CE8	43.027	80.0709	18.1287	25.6374	Pectin methylesterase (EC 3.1.1.11).
CE9	294.834	212.118	474.72	395.33	N-acetylglucosamine 6-phosphate deacetylase (EC 3.5.1.25); N-acetylgalactosamine-6-phosphate deacetylase (EC 3.5.1.80).
CE10	596.732	1009.64	225.639	135.508	Arylesterase (EC 3.1.1.-); carboxyl esterase (EC 3.1.1.3); acetylcholinesterase (EC 3.1.1.7); cholinesterase (EC 3.1.1.8); sterol esterase (EC 3.1.1.13); brefeldin A esterase (EC 3.1.1.-).
CE11	376.726	322.36	228.196	301.047	UDP-3-0-acyl N-acetylglucosamine deacetylase (EC 3.5.1.-).
CE12	98.2928	251.826	203.141	122.558	Pectin acetylesterase (EC 3.1.1.-); rhamnogalacturonan acetylesterase (EC 3.1.1.-); acetyl xylan esterase (EC 3.1.1.72)
CE14	46.9864	308.344	-	-	N-acetyl-1-D-myo-inosityl-2-amino-2-deoxy-alpha-D-glucopyranoside deacetylase (EC 3.5.1.89); diacetylchitobiose deacetylase (EC 3.5.1.-); mycothiol S-conjugate amidase (EC 3.5.1.-)
CE15	5.14113	25.4286	-	-	4-O-methyl-glucuronoyl methylesterase (EC 3.1.1.-)
CE16	22.5685	51.4155	-	-	Acetylesterase (EC 3.1.1.6) is active on various carbohydrate acetyl esters
Others	20,856.9	26,276.4	21,728.5	25,421	-

## Data Availability

Data are contained within the article. The datasets of metagenomic and metatranscriptomic were deposited into the NCBI Sequence Read Archive (SRA), with accession numbers of PRJNA908247 and PRJNA908248, respectively.
